# A Chip Digital PCR Assay for Quantification of Common Wheat Contamination in Pasta Production Chain

**DOI:** 10.3390/foods9070911

**Published:** 2020-07-10

**Authors:** Caterina Morcia, Raffaella Bergami, Sonia Scaramagli, Roberta Ghizzoni, Paola Carnevali, Valeria Terzi

**Affiliations:** 1 Council for Agricultural Research and Economics, Research Centre for Genomics and Bioinformatics, Via San Protaso 302, I-29017 Fiorenzuola d’Arda PC, Italy; caterina.morcia@crea.gov.it (C.M.); roberta.ghizzoni@crea.gov.it (R.G.); 2 Coop Italia, Via del Lavoro, 6/8, I-40033 Casalecchio di Reno BO, Italy; raffaella.bergami@coopitalia.coop.it (R.B.); sonia.scaramagli@coopitalia.coop.it (S.S.); 3 Barilla S.p.A., Via Mantova 166, I-43122 Parma PR, Italy; paola.carnevali@barilla.com

**Keywords:** pasta, *Triticum aestivum*, *Triticum durum*, genetic traceability, digital PCR, semolina, species

## Abstract

Pasta, the Italian product par excellence, is made of pure durum wheat. The use of *Triticum durum* derived semolina is in fact mandatory for Italian pasta, in which *Triticum aestivum* species is considered a contamination that must not exceed the 3% maximum level. Over the last 50 years, various electrophoretic, chemical, and immuno-chemical methods have been proposed aimed to track the possible presence of common wheat in semolina and pasta. More recently, a new generation of methods, based on DNA (DeoxyriboNucleic Acid) analysis, has been developed to this aim. Species traceability can be now enforced by a new technology, namely digital Polymerase Chain Reaction (dPCR) which quantify the number of target sequence present in a sample, using limiting dilutions, PCR, and Poisson statistics. In our work we have developed a duplex chip digital PCR (cdPCR) assay able to quantify common wheat presence along pasta production chain, from raw materials to final products. The assay was verified on reference samples at known level of common wheat contamination and applied to commercial pastas sampled in the Italian market.

## 1. Introduction

Pasta production is a strategic chain in the Italian agri-food sector, covering around the 6% of total industrial output [1]. Italy is at the same time the world’s leading pasta producer, with an annual production around 3.2 million tons and, in the same time, is the largest consumer of pasta (26 kg per capita). A pillar of Italian pasta production chain is the grain identity: The use of *Triticum durum* derived semolina is in fact mandatory for Italian pasta, in which *Triticum aestivum* species is considered a contamination that must not exceed the 3% maximum level, as indicated by Law n.580 of 1967 [2] and by subsequent Decreto del Presidente della Repubblica (D.P.R.) 187, 9 February 2001 [3] and D.P.R. 41, 5 March 2013 [4]. Traditional Italian pasta, according to such regulations, is therefore the result of the extrusion, rolling and drying of dough made exclusively from durum wheat and water. The choice of *Triticum durum* is based on its peculiarities, among others the hardiness of the caryopsis, the intense yellow color due to carotenoids, the gluten composition. Thanks to such specific properties, starch is not lost during cooking, avoiding sticking and ensuring a unique and authentic taste to pasta.

Beyond fraudulent behavior, dictated by the lower price of common wheat compared to durum, the purity of the semolina can also be compromised during the various processing stages of the supply chain, which range from harvesting in the field to storing the grains. Analytical methods have been proposed aiming at the detection and quantification of the possible presence of common wheat in semolina and pasta. In this perspective, over the last 50 years, various electrophoretic, chemical and immuno-chemical methods have been proposed aimed at detecting the purity of the semolina [5,6,7,8,9]. Such methods are based on the identification and quantification of specific protein, which, however, can be degraded by the high temperatures nowadays used to dry pasta. To overcome this gap and taking advantage of the remarkable thermic stability of DNA (DeoxyriboNucleic Acid), a new generation of methods, based on DNA analysis, has been developed during the last two decades. PCR (Polymerase Chain Reaction) based assays to identify common wheat by distinguishing it from durum one has been developed by Bryan et al. [10], by Arlorio et al. [11] and by Sonnante et al. [12], using respectively *Dgas44* gene sequence, puroindoline B and SSR (Simple Sequence Repeats) related sequences. Untargeted DNA fingerprinting through tubulin-based polymorphism (TBP) have been optimized by Casazza et al. [13] and by Silletti et al. [14] for the authentication of cereal species, including wheat and farro. qPCR assays for the quantification of *Triticum aestivum* species have been proposed by Alary et al. [15], Terzi et al. [16], Matsuoka et al. [17], and by Imai et al. [18]. These two last assays have been in-house verified and compared by Paterno’ et al. [19], with the aim to select a taxon-specific assay useful for unauthorized GM (Genetically Modified) wheat detection in wheat samples. An inter-laboratory validation in collaboration with public and private laboratories has been even reported by Morcia et al. [20] to determine the performance parameters of a qPCR assay based on the primers designed on puroindoline-b gene by Alary et al. [15] and on low molecular weight glutenin encoding sequence by Terzi et al. [16].

Species traceability can be now enforced by a new technology, namely digital PCR (dPCR) which quantify the number of target sequence present in a sample, using limiting dilutions, PCR and Poisson statistics [21]. The PCR mix is compartmentalized across a large number of partitions or droplets containing zero, one or more copies of the target sequence. After endpoint PCR amplification, a partition can be positive (‘‘1′’, the presence of PCR product) or negative (‘‘0′’, the absence of PCR product). The absolute number of target nucleic acid molecules contained in the original sample before partitioning can be calculated directly from the ratio of the number of positive to total partitions, obtained using Poisson statistics. It is an absolute quantification strategy because there is not the need to have a standard curve as reference for quantification. In the past several years, dPCR has achieved progress in in agri-food sector, especially for GMO (Genetically Modified Organism) testing [21,22] and for pathogen diagnostics and, at more limited extent, to the detection of animal- and plant-derived ingredients in food adulteration control [23].

The aim of this work has been to develop a chip digital PCR (cdPCR) assay able to quantify common wheat presence along pasta production chain, from raw materials to final products. The assay was verified on reference samples at known level of common wheat contamination and applied to commercial pastas sampled in the Italian market.

## 2. Materials and Methods

### 2.1. Mono-Species Flour Samples Preparation and DNA Extraction

Certified *Triticum durum* (Claudio variety) and *Triticum aestivum* (Eureka variety) seeds were obtained from CREA DC (Tavazzano, Italy). Such first-reproduction seeds are controlled and certified both at species and variety levels. In major details, at species purity level, the maximum admitted contamination is of 7 seeds belonging to different cereal species/500 g of certified seeds, according to the Italian D.P.R. n. 1065, 8 October 1973. The seeds were milled using a Cyclotec (FOSS Italia S.r.l., Padova, Italy) at 0.2 mm grid diameter, avoiding any contamination between samples. Samples of 100% durum wheat semolina and 100% common wheat flour were separately stored at controlled temperature and humidity conditions until further use.

DNA were extracted from three biological replicates of milled *Triticum aestivum* and *Triticum durum* seeds using the DNeasy mericon Food Kit (Qiagen, Milan, Italy), that is based on an improved cetyltrimethylammonium bromide (CTAB) extraction of total cellular nucleic acids. The flour samples (2 g) were extracted according to manifacturer’s instructions. The evaluation of quality and quantity of extracted DNA was done using Qubit™ fluorometer in combination with the Qubit™ dsDNA BR Assay kit (Invitrogen by Thermo Fisher Scientific, Monza, Italy).

### 2.2. Mixed Species DNA Samples Preparation

*Triticum aestivum* and *Triticum durum* DNA, extracted from the mono-species flours described in point 2.1, were mixed to obtain the following samples:

- *T.durum* DNA 99.7% + *T.aestivum* DNA 0.3%;
- *T.durum* DNA 98.5% + *T.aestivum* DNA 1.5%;
- *T.durum* DNA 97% + *T.aestivum* DNA 3%;
- *T.durum* DNA 95.5% + *T.aestivum* DNA 4.5%;
- *T.durum* DNA 70% + *T.aestivum* DNA 30%

### 2.3. Mixed Species Flour Samples Preparation and DNA Extraction

Common wheat flour was used to contaminate durum wheat semolina with the aim to produce durum wheat samples containing 0.3, 1.5, 3, 4.5, and 30% of common wheat. After weighing the common and durum wheat flour, samples containing different percentages of the two species were homogenized for 10 min. DNA were extracted from flours (2 g) with the DNeasy mericon Food Kit (Qiagen, Milan, Italy), as previously described. The evaluation of quality and quantity of extracted DNA was done using Qubit™ fluorometer in combination with the Qubit™ dsDNA BR Assay kit (Invitrogen by Thermo Fisher Scientific, Monza, Italy).

### 2.4. Reference and Commercial Pasta Samples and DNA Extraction

Four reference pasta samples were prepared by mixing tap water and wheat flours containing the following common wheat percentages: 1.5%, 3%, 4.5%, 10%. The samples were dried in oven at 80 °C for 1 hour, followed by 3 hours at decreasing temperature. Such desiccation thermal profile is those commonly used for commercial pasta preparation. DNA were extracted from two biological replicates of reference pasta using the DNeasy mericon Food Kit (Qiagen, Milan, Italy), Twenty commercial pasta samples of different brands were purchased from the market. The pasta samples were milled with M20 Universal Mill (IKA). Samples (2 g) were extracted in single replicate with the DNeasy mericon Food Kit (Qiagen, Milan, Italy), as previously described. The DNA obtained was measured using Qubit™ fluorometer in combination with the Qubit™ dsDNA BR Assay kit (Invitrogen by Thermo Fisher Scientific, Monza, Italy).

### 2.5. Primers and Probes

Primers and probes (Table 1) were designed using Primer Express 3.0.1 Software (Life Technologies Corporation). Each primer was checked for absence of self-complementarity and primer dimer formation with other primer pairs using the online tool Multiple Primer Analyzer (Thermo Fisher Scientific, Monza, Italy). Primer specificity was checked by blasting in EnsemblPlants (https://plants.ensembl.org/index.html) against the *Triticum aestivum* database.

### 2.6. Real-Time PCR

The reaction mixture was prepared in a final volume of 25 µL consisting of 12.5 µL of SYBR Green PCR, 2× GoTaq qPCR Master Mix (Promega Italia, Milan, Italy), 0.25 µl of 100× Reference Dye (Promega Italia, Milan, Italy), 0.5 µL of each primer at 10 µM (final concentration 200 nmol), 4 µL of DNA template serial dilution (10, 5, 2.5, 0.5, 0.25 and 0.025 ng/µL) and water to 25 µL. Three technical real-time PCR replicates were done for each sample and control. The PCR mixture was activated at 95 °C for 10 min. Forty amplification cycles were carried out at 95 °C for 15 s followed by 60 °C for 1 min. A melting curve analysis was included in each run.

### 2.7. Chip Digital PCR

Chip digital PCR was performed using QuantStudioTM 3D Digital PCR System (Applied Biosystems by Life Technologies, Monza. Italy). The reaction mixture was prepared in a final volume of 16 µLconsisting of 8 µL QuantStudioTM 3D Digital PCR 2X Master Mix, 0.72 µL of each primer at 20 µM (final concentration 900 nmol), 0.32 µL of FAM and VIC-MGB probes at 10 µM (final concentration 200 nmol), 2 µl of DNA (40 ng/µL) and nuclease free-water. Also, a negative control with nuclease free-water as template was added. A total volume of 15 µL reaction mixture was loaded onto the QuantStudioTM 3D Digital PCR chips using QuantStudioTM 3D Digital chip loader, according to manufacturer protocol. Amplifications were performed in ProFlexTM 2Xflat PCR System Thermocycler (Applied Biosystems by Life Technologies, Monza, Italy) under the following conditions: 96 °C for 10 min, 45 cycle of 55 °C annealing for 2 min and 98 °C denaturation for 30 s, followed by 60 °C for 2 min and 10 °C. End-point fluorescence data were collected in QuantStudioTM 3D Digital PCR Instrument and files generated were analyzed using cloud-based platform QuantStudioTM 3D AnalysisSuite dPCR software, version 3.1.6. Each sample was analyzed in triplicate.

### 2.8. Triticum aestivum Percentage Calculation

For the common wheat percentage calculation, we start from the absolute copies/µL yielded by the QuantStudioTM 3D Analysis Suite dPCR software. In our assay the *T. aestivum* target sequence is marked with FAM, whereas the taxon target sequence is marked in VIC. Equation 1 was used to calculate the percentage of common wheat copies in the sample, in which FAM stands for the number of FAM copies/µL and VIC for the number of VIC copies/µL:

(1) $$\frac{FAM}{\frac{VIC-3*FAM}{2}+FAM}*100$$

## 3. Results

### 3.1. Reference Samples

Several factors are important for accurate quantification of multiplexed assays, including target linkage, probe specificity and differential PCR efficiencies.

The absence of linking between the two targets has been evaluated through literature and bioinformatic analysis. Nemoto et al. [24] demonstrated, through Southern blot analysis, that the *Triticum TaHd1* gene is present in single copy on each A, B and D genomes of wheat and maps on long arm of chromosome 6. *Pinb-D1*gene maps in D sub-genome and is located on chromosome 5 at the Hardness (Ha) locus. The two targets are therefore not linked.

Primers/probes specificity have been preliminarily evaluated in qPCR, finding that TritA_APX assay gives a signal only in hexaploid wheat, whereas GranoCO2 assay gives a signal both in hexaploid and tetraploid wheats (including farro dicoccum and Kamut).

**Figure 1:**
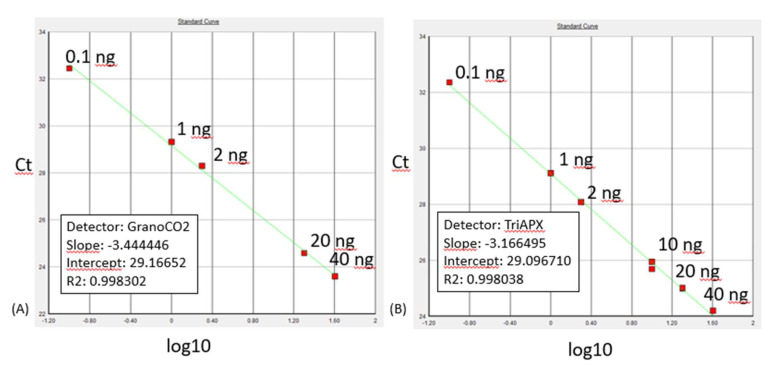
qPCR standard curves obtained after amplification of the DNA dilutions reported in the graph with GranoCO2 primers (**A**) and with TriAPX primers (**B**).

Amplification efficiency and reproducibility for each primer set were examined through a standard curve qPCR assay, using bread and durum wheat DNA dilutions (Figure 1). Efficiency of reactions were calculated from the slope using the formula E  =  10−1/slope. The slope values obtained were of −3.44 for GranoCO2 primers, and of −3.17 was obtained for TritAPX primers. Amplification efficiencies were of 99.6 and 104%, respectively.

**Figure 2:**
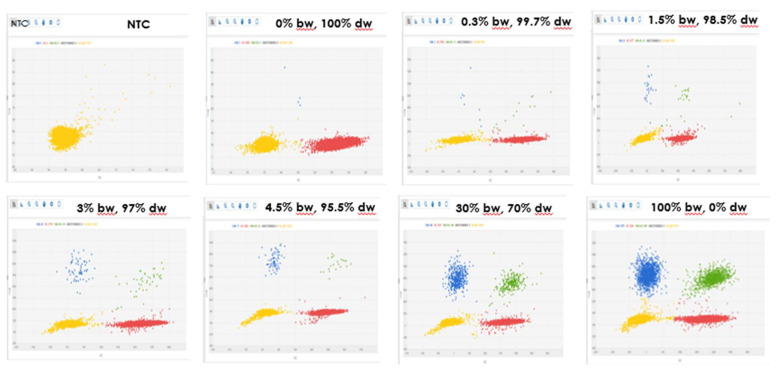
Two-dimensional scatter graphs generated by chip digital PCR (cdPCR) analysis of eight different samples. NTC (No Template Control) is a blank sample without DNA; The other samples are made of durum wheat (dw) DNA or common wheat (bw) DNA or a mix of the two, as indicated in the figure; In this graph a partition can fall into one of four possible clusters: negative partition that contain no amplified targets (yellow), single positive partition for *Triticum* genus (red), single positive partition for common wheat (blue) and positive partitions that contain a positive signal for both targets (green, double-positive partitions).

The duplex method was then optimized in cdPCR system for specificity on the reference samples described in Materials and Methods. The concentrations of primers and probes were optimized at 900 nmol and 200 nmol respectively and the annealing temperature was fixed at 55 °C. The resolution of the clusters (Figure 2) was obtained in absence of restriction digestion of the samples, therefore this time-consuming procedure was omitted from the protocol.

The mean common wheat percentages experimentally determined in “mixed flour” and “mixed DNA” samples in comparison with actual percentages are reported in Table 2. The SD values reported in the same table express the precision of the method, i.e., the closeness of agreement between replicate measurements. At 3% level, the SD values are <35% for all the samples and therefore the precision is acceptable, according to Codex Alimentarius Commission/Guidelines 74–2010 [25]. In Table 2 are even reported some values informative about the precision and the accuracy of the method, such as the coefficient of variation (CV), the absolute error and the relative error.

The trueness of the method is usually defined as the degree of agreement of the expected value with the true value or accepted reference value. In GMO testing the trueness must be within 25% of the accepted reference value [25]. The trueness of our method fits the purpose: The estimated concentrations over the dynamic range tested were within the ± 25% acceptable bias as recommended by GMO analytical guidelines [26]. In particular, at 3% level the experimentally determined percentages are very close to the true one. In the evaluated dynamic range, the LOD (Limit of Detection) of the method has been found at 0.3% common wheat contamination, whereas the LOQ (Limit of Quantification) at 1.5% level.

The Pearson’s r between the expected and calculated common wheat percentages were determined in mixed DNA samples and in mixed flour samples. The correlation values found are respectively of 0.9985 and of 0.9993. Extracting DNA from mixed flours and their subsequent amplification is much more realistic model of real foods, rather than mixing DNA from different species/samples. However, the preparation of mixtures of flours can be potentially affected by weighting errors and by heterogeneity problems, due, for example, to variation in granulometry, in mixing and blending. On the other hand, DNA mixtures can be affected by errors in DNA quantification and mixing. Therefore, with the intent to minimize the inaccuracy of the reference materials we decided to prepare two series of blends using the two different options. After analyses, the two classes of reference materials gave the same results. No statistically significant differences were found among mean common wheat % values determined from mixed DNA samples and from mixed flours. It is therefore possible to conclude that the two classes of reference materials prepared worked in agreement.

Since 3% common wheat threshold is in percentage of mass ratio (% *m/m*) and since the analytical output is in number of common wheat and taxon target copies, a conversion factor is needed. This conversion factor, CF, mainly depends on the zygosity, but even on differences linked to DNA extraction and varieties. CF for GMO detection is available for each CRM (Certified Reference Material) [21].

For our homozygous samples, for which certified reference materials are not available, a conversion from % (copy/copy) to % (m/m) can be hypothesized. This same approach has been used in the study of Dong et al. [23] aimed to quantify kidney bean in lotus seed paste.

In 3% common wheat reference samples, a mean percent recovery of 100.44 has been obtained, that fully fits with the acceptable range for major components in low complexity matrices (95–105%).

### 3.2. Reference and Commercial Pasta Samples

The applicability of duplex dPCR assay to pasta was evaluated in two different groups of samples: 4 reference pasta samples prepared in our laboratory and contaminated with 1.5%, 3%, 4.5%, and 10% common wheat and on 20 pasta samples of different brands commercialized in Italy.

**Figure 3:**
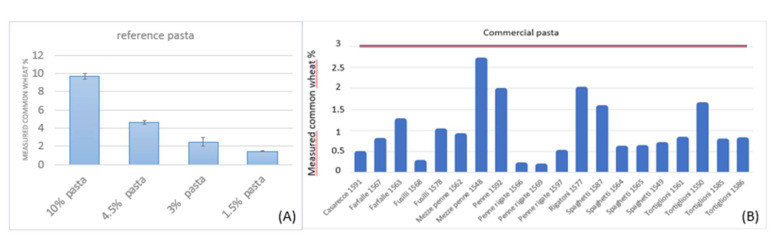
Common wheat percentages determined in 4 reference pasta (**A**) and in 20 commercial pasta samples (**B**) with duplex digital PCR (dPCR) assay. In (A) the percentages values before the word “pasta” indicate the common wheat contaminations. In (B) the red horizontal line indicates the maximum level of common wheat contamination allowed by law.)

The results are reported in Figure 3, from which it can be observed that the duplex dPCR assay performs well on reference pasta, with a correlation value of 0.99 among actual and measured percentages and a mean relative error of 0.07.

As previously introduced, a body of Italian laws and regulations rule the product named “pasta” [2,3,4]. The denomination “pasta” strictly defines a product obtained after drawing, rolling and subsequent drying of a dough exclusively made from durum wheat (flour or semolina or whole semolina) and water. In the final product the humidity must not exceed 12.50%. The production of pasta with common wheat flour is forbidden, but a maximum level of 3% common wheat flour is tolerated as result of accidental contamination during the production chain. The inclusion of ingredients different from durum wheat and water is reserved to “special pasta”. The special pastas must be offered for sale in Italy with the name durum wheat semolina pasta supplemented by the mention of the ingredient used and, in the case of several ingredients, of that or the characterizing ones. Anyway, even in special pasta, common wheat is a contaminants. The special pasta represents a minor sector of Italian pasta production and consumption. Therefore, as representative of the market, pasta of different brands has been considered in this study. The analyzed samples were all labelled as “pasta” and all reported, as ingredients, durum wheat and water. According to Italian laws, a maximum 3% common wheat presence is expected. All the commercial samples have been found below the 3% common wheat contamination threshold. The analytical data confirm that all the samples comply with the Italian laws.

## 4. Discussion

We have developed a duplex chip digital PCR analytical protocol to identify and quantify common wheat contamination in pasta production chain. The reason for developing such new assay is related to dPCR particularities. In comparison with conventional end-point PCR and qPCR, this technique has been reported to have many advantages (reviewed by Demeke et al. [27]), the major the absolute quantification of a target without reference to a standard/calibration curve. This fact reduces the errors deriving from the comparison of different matrices, i.e., the calibrant and the test sample. Moreover, because of the high-level sample partitioning, dPCR is less sensitive to PCR inhibitors and the results obtained are potentially very precise and accurate [27,28]. Thanks to the high resilience to inhibitors, the efficiency and the reproducibility on different platforms, dPCR is candidate as higher-order reference measurement methods and as the method for value assignment of reference materials [28]. On the other hand, a limitation of such approach is that it is more expensive than qPCR, but the use of multiplex approaches moves the scales in favor of dPCR [27]. From a technology transfer point of view, both the pasta industry and the large consumer cooperative, between the other involved in this work, expressed interest in developing and applying a dPCR strategy for control pasta chain. The key control points are in the passage of the grains from stackers to the mills, of semolina batches from the mills to the pasta factory and in the final product, the pasta. The pasta chain stakeholders interested in such analytical tool are therefore the farmer associations, the stackers, the mills, the pasta industry, the consumer associations and the public and private control bodies. All the stakeholders have the interest to share a method for common wheat contamination control in grains, semolina, and pasta. Several assays has been developed and validated for such purpose, but are all dependent on a calibration curve and suffer from the loss of certified reference materials for the construction of such curves. DigitalPCR, that works without the need of calibrants, can fill this gap. It can in fact be proposed as method for the validation of reference materials to be used for qPCR standard curves and as higher order reference measurement method. This hypothesis to apply dPCR technology to prepare reference materials has been advanced by other authors, e.g., Mehle et al. [29] in plant pathogen detection, by Dong et al. [30] in environmental microbiology and by Pavšič et al. [31] in microbial diagnostics. The potential for synergy of qPCR and dPCR has been underlined by Debski et al. [32]. in the field of medical diagnostics. In conclusion, the opportunity to complement and strengthen the cheaper qPCR analyses justify the higher cost of dPCR assays.

Our cdPCR assay is based on duplex non-competing reactions: two amplicons are generated from two primer sets and the signal generated from a probe specific for each amplicon enable to distinguish the two targets within a single reaction. Such concurrent amplifications reduce technical errors, reagent and time needed. One of the target is a D-genome specific genic sequence and the other a *Triticum* specific genic sequence present in A, B and D genomes. This taxon-specific assay was designed on *TaHd1* gene sequence. Such gene, involved in the photoperiodic flowering pathway, has been demonstrated to be present in single copy in each of the A, B and D *Triticum* genomes [20]. The bread wheat specific assay was designed on *Pinb-D1*, a single-copy gene encoding for puroindoline b protein [15,33]. This gene belongs to the Ha locus, occurring only on chromosome 5D in common wheat [26]. Accordingly, we have developed the formula reported in Materials and Methods for the common wheat % calculation. In the formula we have considered:

- The different level of ploidy between common wheat (hexaploid, with the three A, B and D genomes) and durum wheat (tetraploid, with A and B genomes);
- the fact that *TaHd1* gene is present in single copy/A, B and D haploid genomes;
- the fact that *Pinb-D1* gene is present in single copy/D haploid genome; and
- the comparable amplification efficiency of the two targets

The *Pinb-D1* gene sequence has been used to target common wheat in cqPCR assays previously developed, whereas the *TaHd1* gene sequence has never been used in pasta authenticity assessment.

As verified on reference samples, the proposed protocol highly performs to track 3% common wheat contamination, that is the critical value fixed by law as limit between accidental contamination and fraud. Its applicability has been evaluated on reference and commercial pasta samples. In conclusion, a cdPCR duplex assay has been developed to control pasta production chain from an economically motivated adulteration, that is the use of cheaper ingredient (i.e., common wheat) instead of durum wheat for pasta manufacturing. It is possible to quantify the mass of common wheat directly in flours and in highly processed food, such as pasta. The inter-laboratory validation of the method can be proposed as further step.

## Figures and Tables

**Table 1:** Primers and probes.

Name	Primer Sequence (5′-3′)	Gene	Target
GranoCO2- Forward	TGCTAACCGTGTGGCATCAC	*Triticum TaHd1*	*Triticum genus*
GranoCO2 Reverse	GGTACATAGTGCTGCTGCATCTG		
GranoCO2 probe	VIC- CATGAGCGTGTGCGTG -MGB		
TritA_APX Forward	AGGAGCGGCCGAAGCT	*Pinb-D1*	*Triticum aestivum*
TritA_APX Reverse	TGTGAAACATCGCTCCATCAC		
TritA_APX probe	FAM-AGCTCTTGCAAGGAT -MGB		

**Table 2:** Actual common wheat percentages in comparison with those experimentally determined in two different classes of samples. “Mixed DNA” samples were obtained by mixing DNA extracted from pure common and durum wheat species. “Mixed flour” samples were obtained by extracting DNA from of common and durum wheat flours mixed at different percentages). CV: Coefficient of variation.

Actual Common Wheat %	Mixed DNA					Mixed Flour				
	Mean Common Wheat %	Std Dev	CV	Absolute Error	Relative Error	Mean Common Wheat %	Std Dev	CV	Absolute Error	Relative Error
0	0.12	0.05	0.39	0.12		0.09	0.06	0.65	0.09	
100	105.00	7.00	0.07	5.00	0.05	94.40	6.85	0.07	5.60	0.06
0.3	0.43	0.05	0.12	0.13	0.43	0.37	0.12	0.34	0.07	0.23
1.5	1.37	0.07	0.05	0.13	0.09	1.43	0.28	0.19	0.07	0.05
3	3.06	0.05	0.01	0.06	0.02	2.86	0.32	0.11	0.14	0.05
4.5	4.50	0.04	0.01	0.00	0.00	3.93	0.51	0.13	0.57	0.13
30	25.90	0.46	0.02	4.10	0.14	24.90	1.68	0.07	5.10	0.17
